# Machine learning enabled classification of lung cancer cell lines co-cultured with fibroblasts with lightweight convolutional neural network for initial diagnosis

**DOI:** 10.1186/s12929-024-01071-0

**Published:** 2024-08-23

**Authors:** Adam Germain, Alex Sabol, Anjani Chavali, Giles Fitzwilliams, Alexa Cooper, Sandra Khuon, Bailey Green, Calvin Kong, John Minna, Young-Tae Kim

**Affiliations:** 1 Department of Bioengineering, University of Texas at Arlington, 500 UTA Blvd ERB244, Arlington, TX 76010 USA; 2 Department of Computer Science, University of Texas at Arlington, Arlington, TX USA; 3 Department of Biology, University of Texas at Arlington, Arlington, TX USA; 4 Department of Nursing, University of Texas at Arlington, Arlington, TX USA; 5 Hamon Center for Therapeutic Oncology Research, University of Texas Southwestern Medical Center, Dallas, TX USA; 6 Department of Urology, University of Texas Southwestern Medical Center, Dallas, TX USA

**Keywords:** Lung cancer, Machine learning, Co-culture, Classification, On-device diagnosis

## Abstract

**Background:**

Identification of lung cancer subtypes is critical for successful treatment in patients, especially those in advanced stages. Many advanced and personal treatments require knowledge of specific mutations, as well as up- and down-regulations of genes, for effective targeting of the cancer cells. While many studies focus on individual cell structures and delve deeper into gene sequencing, the present study proposes a machine learning method for lung cancer classification based on low-magnification cancer outgrowth patterns in a 2D co-culture environment.

**Methods:**

Using a magnetic well plate holder, circular pattern lung cancer cell clusters were generated among fibroblasts, and daily images were captured to monitor cancer outgrowth over a 9-day period. These outgrowth images were then augmented and used to train a convolutional neural network (CNN) model based on the lightweight TinyVGG architecture. The model was trained with pairs of classes representing three subtypes of NSCLC: A549 (adenocarcinoma), H520 (squamous cell carcinoma), and H460 (large cell carcinoma). The objective was to assess whether this lightweight machine learning model could accurately classify the three lung cancer cell lines at different stages of cancer outgrowth. Additionally, cancer outgrowth images of two patient-derived lung cancer cells, one with the KRAS oncogene and the other with the EGFR oncogene, were captured and classified using the CNN model. This demonstration aimed to investigate the translational potential of machine learning-enabled lung cancer classification.

**Results:**

The lightweight CNN model achieved over 93% classification accuracy at 1 day of outgrowth among A549, H460, and H520, and reached 100% classification accuracy at 7 days of outgrowth. Additionally, the model achieved 100% classification accuracy at 4 days for patient-derived lung cancer cells. Although these cells are classified as Adenocarcinoma, their outgrowth patterns vary depending on their oncogene expressions (KRAS or EGFR).

**Conclusions:**

These results demonstrate that the lightweight CNN architecture, operating locally on a laptop without network or cloud connectivity, can effectively create a machine learning-enabled model capable of accurately classifying lung cancer cell subtypes, including those derived from patients, based upon their outgrowth patterns in the presence of surrounding fibroblasts. This advancement underscores the potential of machine learning to enhance early lung cancer subtyping, offering promising avenues for improving treatment outcomes in advanced stage-patients.

**Supplementary Information:**

The online version contains supplementary material available at 10.1186/s12929-024-01071-0.

## Background

Lung cancer classification is an important step in ensuring patients receive the correct treatment, especially as treatments become more customized to individual patient needs. Without accurately identifying the type of tumor, molecular targeted therapies and immunotherapies have significantly reduced efficacy [1–3]. While small cell lung carcinoma (SCLC) and non-small cell lung carcinoma (NSCLC) are typically distinguishable due to the oat-like appearance of SCLC, there exist subtypes within both categories that are more challenging to differentiate. NSCLC subtypes include adenocarcinoma (ADC), squamous cell carcinoma (SCC), and large cell carcinoma (LCC). SCLC encompasses small cell or combined small cell carcinoma types [2, 4, 5]. Current diagnostic methods involve morphological examination, followed by immunohistochemistry and genetic testing [1]. Genetic testing, while crucial, benefits from first identifying the correct cancer subtype to narrow down the specific mutations present in tumor cells. Machine learning-enabled neural networks offer a unique opportunity to detect trends in images reliably, which may not be easily discernible by humans.

Convolutional Neural Networks (CNNs) have been widely utilized in image classification, with models such as AlexNet [6], VGGnet [7], ResNet [8], and DenseNet [9] demonstrating exceptional performance in various image classification competitions. CNNs excel in image classification due to their ability to learn diverse features within images by converting pixels into numerical intensity values and applying mathematical filters to detect specific patterns such as corners and edges, which are critical features for classification tasks. In the domain of lung cancer classification, CNNs have been extensively utilized with various data inputs. At the immunohistochemistry level, several studies have reported CNNs capable of analyzing histopathological slides of lung cancers and achieving classification into subtypes with success rates comparable to or higher than those of trained histopathologists [5, 10–13]. These methods offer significant benefits by providing confirmation or supplementary classification to enhance diagnostic confidence. Additionally, researchers have explored genetic approaches using neural networks, where gene profiles are input into algorithms to predict lung cancer subtypes [2, 14, 15]. These methods enable precise subtype classification, such as distinguishing different adenocarcinoma categories like lepidic (LEP), papillary (PAP), acinar (ACN), micropapillary (MIP), or solid (SOL). Such classifications are pivotal for applying targeted therapies based on identified gene expressions [1, 15, 16]. These CNN-based approaches typically analyze data from a single point in time since cancer cells need to be fixed for histopathological examination and lysed for genetic expression analysis.

In this study, we utilize a machine learning-enabled approach to classify lung cancer subtypes using a lightweight CNN model known as TinyVGG. Our specific focus was on classifying three subtypes of NSCLC: A549 (adenocarcinoma), H520 (squamous cell carcinoma), and H460 (large cell carcinoma). Our objective was to assess the capability of this lightweight model to accurately classify these lung cancer cell lines at various stages of cancer outgrowth. Furthermore, we employed the same lightweight model to classify patient-derived lung cancer cells, both categorized as adenocarcinomas, with distinct genetic profiles: one harboring the KRAS oncogene (HCC 4087) and the other the EGFR oncogene (HCC 4190). This investigation aims to assess the translational potential of machine learning in lung cancer classification. To facilitate this study, we utilized a specialized magnetic well plate holder capable of isolating islands of cancer cells surrounded by fibroblasts. This setup enhances the growth rate of the cancer cells [17] and simulates the cellular interactions, such as invasion and metastasis, between cancerous and healthy cells (Supplementary Fig. 1). Over a period of 9 days, we monitored and captured (and augmented) the outgrowth patterns of cancer cells to generate comprehensive training data for machine learning, encompassing five different lung cancer subtypes.

## Methods

### Cell lines, patient-derived lung cancer cells, culture, and reagents

We obtained primary human dermal fibroblast (HDF-a), NCI-H460 (large cell carcinoma), A549 (adenocarcinoma), and H520 (squamous cell carcinoma) cell lines from ATCC. These cell lines were cultured in RPMI medium supplemented with 10% fetal bovine serum at 37 °C with 5% CO_2_. Patient-derived lung cancer cells, HCC 4087 and HCC 4190, were provided by UT Southwestern Medical Center. Both cells are classified as adenocarcinoma, confirmed via patient-derived xenograft (PDX) model and comprehensive genetic analysis: HCC 4087 harbors the KRAS oncogene, while HCC 4190 carries the EGFR oncogene. These cells were cultured in RPMI medium supplemented with 10% fetal bovine serum. For 3D printing of magnetic cell culture platform, we used Anycubic Photon Mono and Anycubic 3D Printer 405 nm UV-Curing Resin, purchased from Amazon. The convolutional neural network (CNN) was trained on two different on-device platforms without network or cloud connectivity: a laptop equipped with a Nvidia GeForce GTX 1050 with 4 GB of video RAM (Intel i5 9300, 8 GB system RAM) and a laptop equipped with AMD Ryzen 5800HS with 12 GB system RAM.

### Lung cancer and fibroblast co-culture for cancer outgrowth collection and data augmentation for machine learning

#### Magnetic co-culture device preparation and cell seeding

**Fig. 1:**
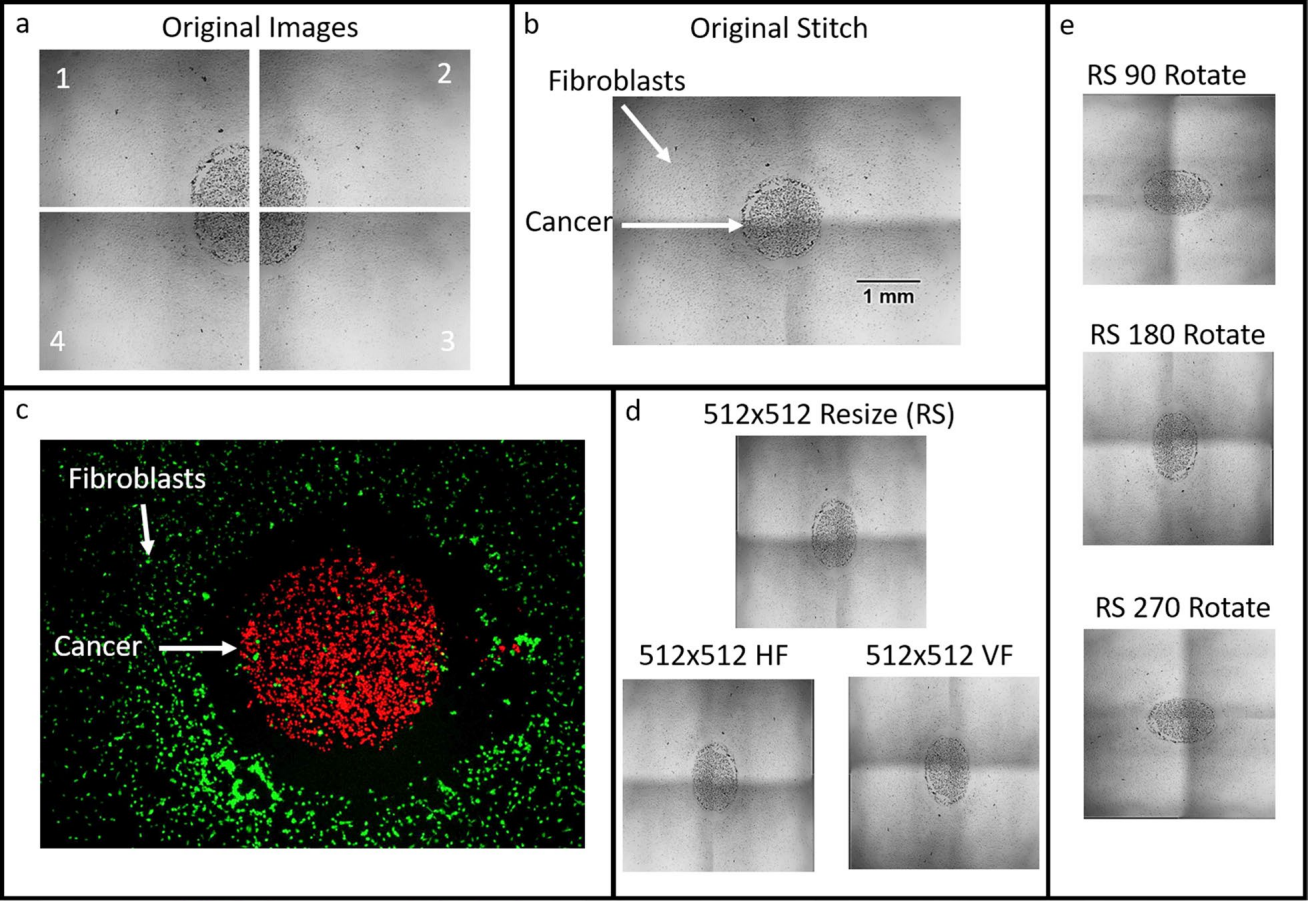
Lung cancer growth collection, image modification, and augmentation for machine training. **a** Original images taken sequentially, as indicated by the numbers in the corner (2 × 2 grid at 2.5 × magnification). **b** Combined image after applying a stitching program. Scale bar = 1 mm. **c** Fluorescent image showing differentially stained cells prior to seeding: cancer cells (H460) in red and healthy HDF-a fibroblasts in green. **d** Original image resized to 512 × 512 pixels (RS) and then augmented by horizontal flipping (HF) and vertical flipping (VF). **e** Examples of original image rotations at 90°, 180°, and 270° for data augmentation

A 12-well magnet holder was created using 3D-printed UV resin. Magnets were inserted into the holder, which was then sterilized. Inside a biosafety cabinet, a 12-well plate was placed atop the magnet holder. Sterilized stainless-steel tubes were positioned over the magnets, within each well [17]. Each well was seeded with 5,000 cancer cells (H460, A549, H520, HCC 4087, or HCC 4190) in 3µL inside the tube and 20,000 HDF-a fibroblasts in 40µL outside the tube. Cells were incubated 37 °C with 5% CO_2_ for 3 h to allow cell attachment to the well plate. After incubation, the tubes were carefully removed using tweezers within the biosafety cabinet. Brightfield images were taken to confirm correct seeding and to record the initial state of each well (Day 0, Fig. 1c).

#### Culturing and imaging

Following the initial imaging, 1.5 mL of fresh RPMI medium supplemented with 10% FBS was added to each well. The cells were incubated for 9 days. Daily images were captured at a consistent time for each well. Images were taken in a 2 × 2 grid at 2.5 × magnification, centered on the seeding island formed by the tube (Fig. 1a). These images were stitched together using the stitching program developed by Preibisch et al. [18]. To ensure consistency, the images were centered and cropped to a uniform area using Adobe Photoshop.

#### Data augmentation for machine learning

To expand the dataset for machine learning, we performed data augmentation on the collected cancer outgrowth images. The initial dataset consisted of images from 86 wells (H460), 42 wells (A549), 44 wells (H520), 37 wells (HCC 4087), and 42 wells (HCC 4190). Due to the low number of images, we augmented the data by performing rotation and mirroring transformations. Each image was resized to 512 × 512 pixels and then flipped horizontally and vertically. The original and transformed images were rotated by 90, 180, and 270 degrees, creating a dataset 12 times larger than the original. This augmentation resulted in the following datasets for machine learning: H460 (8,292 augmented images for training and 1,919 images for validation), A549 (4,200 images for training and 839 images for validation), H520 (4,308 images for training and 959 images for validation), HCC 4087 (3,564 images for training and 792 images for validation), and HCC 4190 (3,372 images for training and 696 images for validation). The purpose of this extensive data augmentation was to enhance the generalization capability of the convolutional neural network (CNN) model, allowing it to better predict and forecast outcomes with new data (i.e., unseen images for validation). The chosen augmentations were justified by the radial and random outgrowth patterns of the cancer cells, ensuring that rotated configurations would still reflect natural cancer outgrowth behaviors (Fig. 1).

### TinyVGG convolutional neural network parameters for cancer classification

**Fig. 2:**
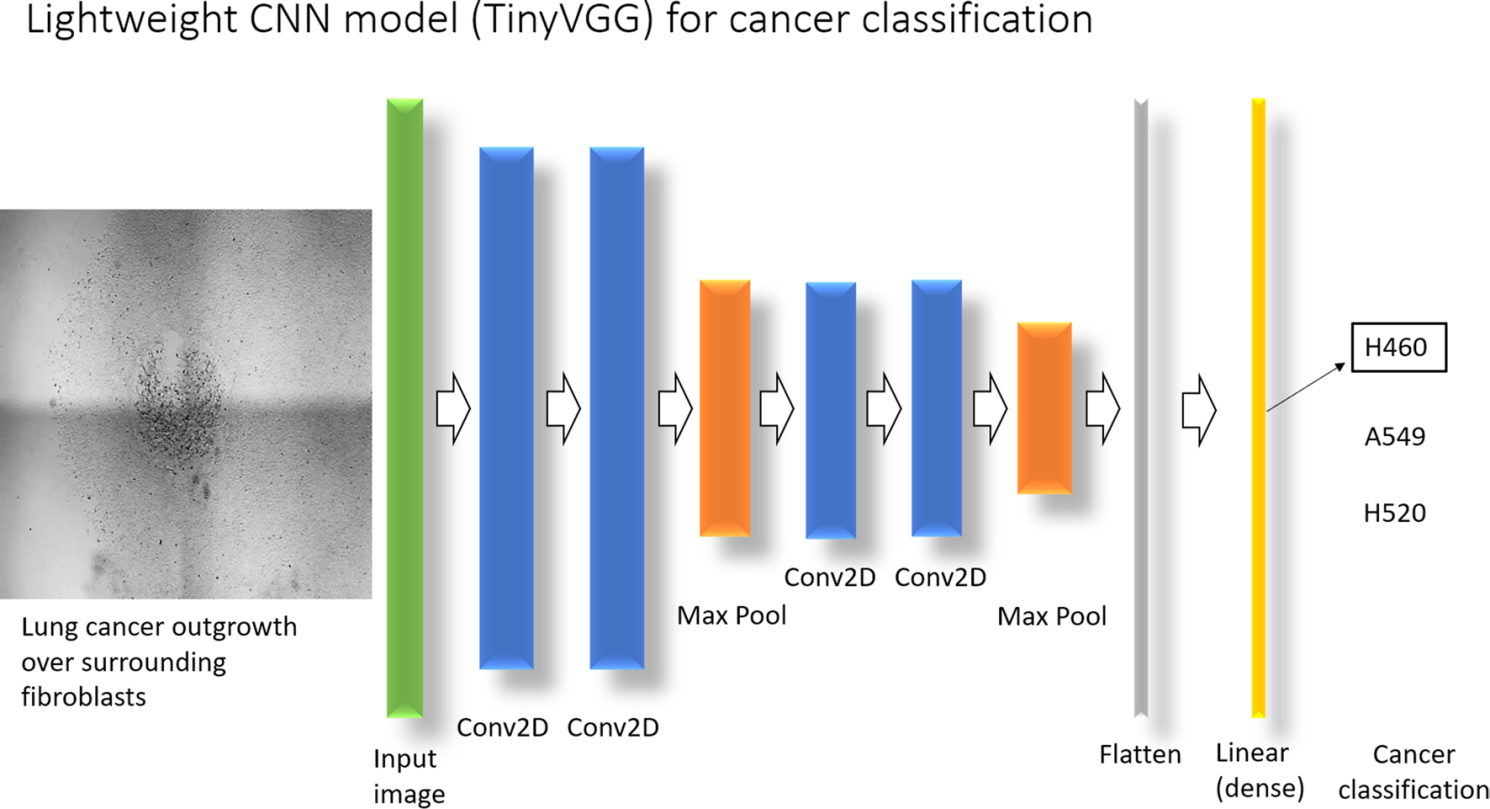
Schematic of the TinyVGG model architecture for lung cancer classification. The TinyVGG model for lung cancer classification is initialized with random weights. Training images, depicting lung cancer outgrowth over surrounding fibroblasts (e.g., 8292 augmented H460 images for training), are processed in batches over a designated number of epochs. The batch size refers to the number of images processed before updating the model’s weights. An epoch represents a complete pass through the entire training dataset. After each epoch, the validation set (e.g., 1919 augmented H460 images for validation) is used to assess the model’s accuracy on unseen data (Validation accuracy). The model iteratively optimizes its weights to minimize the training loss. The architecture includes multiple layers, with Conv2D denoting 2D Convolution layers

The most basic structure of a CNN model includes the following layers, with the output of each layer feeding into the next: a convolutional layer, an activation function, a pooling layer, a flattening layer, a dense layer, and a loss function. We used the lightweight TinyVGG model [19] (shown in Fig. 2) because it is designed for image classification tasks while requiring fewer computational resources. This allows it to operate locally on a secured laptop without network or cloud connectivity and simplifies training by having fewer parameters to adjust.

#### Convolution

The convolutional layers have customizable parameters such as output-channels, kernel size, stride length, and padding. These parameters create a number of filters equal to the input channels multiplied by the output channels, with a size determined by the kernel size, stride length, and padding. The kernels perform mathematical operations on the pixels using a matrix the size of the kernel dimensions. The kernel operates on a set of pixels and translates across the image by a number of pixels based on the stride value. Padding adds a layer of blank pixels around the edge of the image to retain image size and enhance edge effects. Without padding, the edges of an image would be reduced due to fewer kernel operations applying to them.

For example, a 512 × 512x1 pixel image input with 3 output channels, a kernel size of 3 × 3 pixels, a stride of 1 pixel, and padding of 1 pixel generates an output of 512 × 512x3. Changing the kernel size to 4 × 4 pixels and the stride to 2 pixels results in an output feature map of 256 × 256 pixels, halving the width and height dimensions of the output tensor. The feature maps are learned based on the model’s loss function, with more feature maps meaning more trainable parameters.

In our model, the parameters of kernel size, stride length, padding, and filters for the convolutional 2D layers are shown in Table 1. The Kernel size was chosen to give the largest feature maps. Layer 1 (first Conv2D in Fig. 2, first blue bar) uses a kernel size, stride, and padding to reduce image size by half (from 512 × 512x1 to 256 × 256x1) for model complexity reduction, while Layers 2–4 (2nd to 4th blue bars in Fig. 2) maintain the image size between input and output. Further reductions in size between Layer 2 and Layer 3 are due to the Max Pooling functions (orange bar in Fig. 2). Output channels are maintained at 10 to increase filters after the initial convolution (e.g., 10 to 100 filters) to capture higher-order features. H x W x C = Height x Width x Channel(s).

**Table 1:** List of utilized convolutional layer parameters for lung cancer classification

Parameters	Layer 1	Layer 2	Layer 3	Layer 4
Kernel Size	4 × 4	3 × 3	3 × 3	3 × 3
Stride Length	2	1	1	1
Padding	1	1	1	1
Filters	10	100	100	100
Input Size (HxWxC)	512 × 512x1	256 × 256x10	128 × 128x10	128 × 128x10
Output Size (HxWxC)	256 × 256x10	256 × 256x10	128 × 128x10	128 × 128x10

#### Activation

The activation function applies an element-wise operation to the feature maps of the convolutional layer and always follows a convolutional layer. Often, the activation function is assumed and not included in the visual representation of a CNN model. Common activation functions include ReLU (rectified linear unit), sigmoid, and hyperbolic tangent functions. The ReLU function sets all negative values to zero while directly transferring the positive values (y = x), and it is the function utilized in our model.

#### Pooling

Pooling layers (orange bars in Fig. 2) are used after convolutional blocks (multiple convolutional layers and activation functions) to reduce the computational load of training and mitigate overfitting. The inputs to a pooling layer are the kernel size and stride. Our model uses a 2 × 2 pixel kernel with a stride of 2, which reduces the output feature map to half the size for both width and height, ending with 25% of the starting data. The two common types of pooling layers are average pooling and max pooling. Average pooling takes the average of the values in the 2 × 2 kernel as the output for a single value, while max pooling takes the highest value of the kernel as the output. Max pooling, which preserves the most prominent features, is the pooling layer utilized in our model.

#### Flatten and dense layers

The flatten layer (gray bar in Fig. 2) transforms the 3D matrix of data into a 1D vector so that the dense layer (yellow bar in Fig. 2) can utilize the features found and identify patterns for cancer classification. The dense layer creates the parameters to be adjusted for the resulting output classification (H460 classification in Fig. 2). We use the Linear function in Pytorch, which creates a model of y = xA^T^ + b, where x is the input value, y is the output value, and A, T and b are learnable weights, for each input value in the flattened tensor. Essentially, each pixel value has a customizable linear function associated with it that the model can tune during training. These sum together to form the overall output classification of the model given an input.

#### Loss function and accuracy

The loss function gauges the effectiveness of the weights in the dense layer. We used the Cross Entropy function, which compares the predicted class of an input image (based on the output of the dense layer) with the ground truth value (label) of the image class. The magnitude of the difference between the true classification value and the output of the algorithm is the “Loss”. The algorithm undergoes training sessions where the input data is fed into the model, and the weights are periodically adjusted to minimize the loss function. Accuracy is another common metric in classification that indicates how often the algorithm correctly classifies images. While accuracy is not related to the magnitude of classification errors, it is useful for diagnosing training issues and interpreting the acceptability of loss values.

#### Training and validation

Typically, the dataset is split for training and validation, with approximately 80% of the data set aside for training and the remainder for validation. The training dataset informs the algorithm’s weights, while the validation dataset serves as the optimization metric. This split helps reduce overfitting, as the validation dataset should be “unknown” to the algorithm regarding weighting and provides initial feedback on the model’s potential efficacy.

## Results

For this lightweight convolutional neural network (CNN), our objective was to determine how effectively the model could classify different lung cancer subtypes. Initially, we conducted tests on H460 outgrowth sequences to evaluate the model’s ability to classify outgrowth of the same cancer type. Although cancer cells generally grow rapidly, there is inherent variability in their growth rates. After these initial tests, we investigated the minimum number of days of outgrowth necessary for the model to accurately classify three subtypes of non-small cell lung cancer (NSCLC): A549 (Adenocarcinoma), H520 (Squamous cell carcinoma), and H460 (Large cell carcinoma). The same days of outgrowth (e.g., Day 0, 1, or 9) for each cancer subtype were classified, aiming to establish a standard incubation period that could be used in the future clinical setting. Additionally, we applied the same lightweight model to classify patient-derived lung cancer cells. These cells, both categorized as adenocarcinomas, had distinct genetic profiles: one harboring the KRAS oncogene (HCC 4087) and the other the EGFR oncogene (HCC 4190). This investigation aims to evaluate the translational potential of machine learning in lung cancer classification.

### Classification of different days of outgrowth within each cell line

**Fig. 3:**
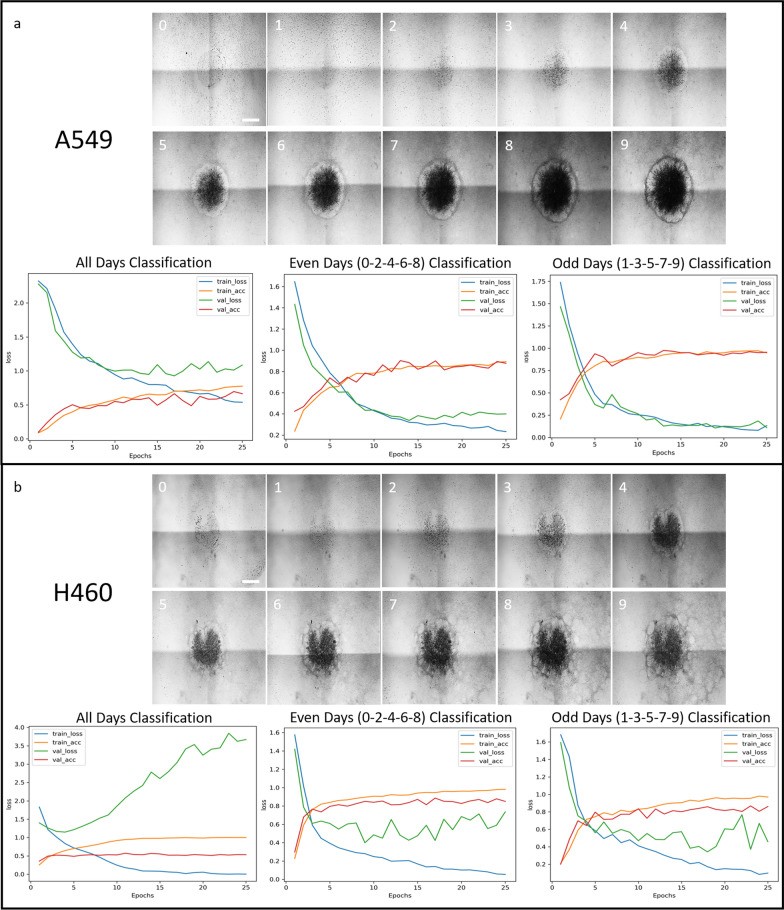
A549 and H460 Individual Time Series Classification. **a** Representative sequences of data for A549 cancer cells over 9 days of outgrowth, with day labels in the corner of each image. Day 0: 3 h after seeing, showing both lung cancer cells at the center and surrounding fibroblasts. Days 1–9: Subsequent days after seeding. The A549 time sequences from Day 0 to Day 9 were trained on the TinyVGG model for 25 epochs (x-axis). **b** Representative sequences of data for H460 cancer cells over 9 days of outgrowth, with day labels in the corner of each image. Day 0: 3 h after seeing, showing both lung cancer cells at the center and surrounding fibroblasts. Days 1–9: Subsequent days after seeding. The H460 time sequences from Day 0 to Day 9 were also trained on the TinyVGG model for 25 epochs. The graphs for both cell lines show training loss (blue), training accuracy (orange), validation loss (green), and validation accuracy (red) achieved across all days (left), even days (middle), and odd days (right). Loss (y-axis) represents the difference between the predicted classification and the true classification. Accuracy is the ratio of correct classifications to total classifications, where an accuracy of 1 indicates 100% correct classification. The model updates its parameters based on the training data and reports the metrics at the end of each epoch. For example, the H460 all-days classification (left-bottom) shows an increasing validation loss, while the other metrics (training accuracy, training loss, and validation accuracy) remain constant as epoch increases. This indicates that the model is making adjustments to improve training loss, but these adjustments result in incorrect classifications for the validation data, leading to higher validation loss without significant improvement in correct classifications. Scale bar = 1 mm

The first test aimed to evaluate how well the model could classify different days (e.g., Days 0–9) of growth for the same type of cancer. If the model struggled with this task, it would indicate that significant differences are needed between days of outgrowth for accurate classification. As seen in Fig. 3a, the most noticeable visual differences between days of cancer outgrowth occurs around days 3–5. By this time, the cancer cells have fully occupied the center starting area and grown in multiple layers, causing significant darkening of the center pixels. An outer ring of slightly darkening pixels also forms, providing two significant features for the model to learn.

The model was trained with a batch size of 128, a learning rate of 0.001, and a weight decay of 0.003 for 25 epochs. The training results for the convolutional neural network are shown in Fig. 3a for A549 and Fig. 3b for H460. The model was trained to classify all days of growth (0–9) for either A549 (Fig. 3a, left-bottom) or H460 (Fig. 3b, left-bottom) and achieved around 50% (0.5 in y-axis) validation classification accuracy. This means that the model has a 50% chance of correctly classifying the level of cancer outgrowth as a specific ‘day’ when given any single day from the entire sequence of outgrowth. For A549 time sequences, the validation loss was around 1, indicating that the model was, on average, accurate within ± 1 day or had difficulty distinguishing between consecutive days of outgrowth. The H460 cells exhibited a continually increasing validation loss (Fig. 3b, left-bottom; green line), suggesting that while the tuning parameters improved the accuracy of correct guesses, the incorrect guesses deviated further from the actual day.

Classifications for every other day were similar for both cancer cell types. For A549 cells, the model achieved validation accuracies of 87.7% for even days (Fig. 3a, middle-bottom) and 95.4% for odd days (Fig. 3a. right-bottom). This indicates that when the difference between days is significant, the model’s certainty increases substantially. For H460 cells, the model achieved validation accuracies of 85.0% for even days (Fig. 3b, middle-bottom) and 86.1% for odd days (Fig. 3b, right-bottom). Although the model performed slightly worse for H460 days of growth, its performance was comparable to the A549 classifications when considering every other day of cancer outgrowth.

This first test suggests that when the differences between lung cancer subtypes are significant, the model may accurately classify the lung cancer subtypes.

### NSCLC subtype classification by day of *cancer* outgrowth

**Fig. 4:**
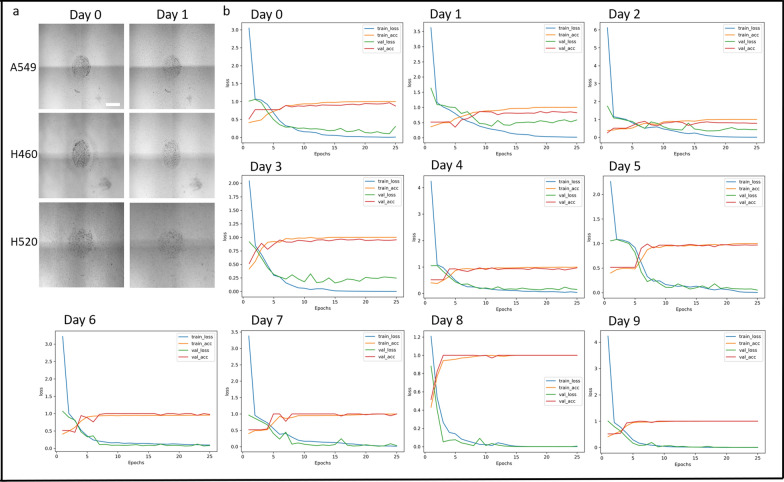
NSCLC subtype classification by day of growth to determine minimum outgrowth for accurate lung cancer classification. **a** Representative images of cancer outgrowth for three NSCLC subtypes (A549, H460, and H520) at Day 0 and Day 1. Scale bar = 1 mm. **b** The three NSCLC subtypes were trained for 25 epochs using the lightweight TinyVGG model. All training parameters were kept consistent across all 10 sets of training (Day 0–9). The same days of outgrowth for each cancer subtype were paired (e.g., Day 1: H460 cancer outgrowth one day after seeding is paired with both A549 and H520 cancer outgrowth one day after seeding) to assess the model’s ability to accurately classify NSCLC subtypes at various stages of outgrowth. The graphs display the training loss (blue), training accuracy (orange), validation loss (green), and validation accuracy (red). The x-axis represents the number of epochs, and the y-axis represents the classification accuracy, where an accuracy of 1 indicates 100% correct classification

Significantly different outgrowth patterns over healthy fibroblasts were observed for three NSCLC subtypes: A549, H460 (Fig. 3), and H520 (Supplementary Fig. 2). For the first few days (Day 0–2), it was challenging to distinguish the three subtypes through visual inspection (Fig. 4a). However, as the cells progressed to Day 9 of outgrowth, the differences became more noticeable. A549 (adenocarcinoma) exhibited a thick, intact contact border throughout the outgrowth over the fibroblasts (Fig. 3a). H460 (large cell carcinoma) formed small individual pockets at its border and more satellite colonies, likely migrating from the main mass of cells created during the initial seeding (Fig. 3b). H520 (squamous cell carcinoma), in contrast to A549 and H460, showed slow growth over fibroblasts without darkening (Supplementary Fig. 2). Days 0–2 showed nearly equal growth rates with a similar pattern of density increase from Day 0 to Day 1, followed by thin border formation and center mass density increase from Day 1 to Day 2. Day 2 to Day 5, H460 cells showed slightly faster boundary formation and darkening of the center cancer compared to the A549 and H520. From Days 5–9, H460 cells lost their distinct round boundary due to smaller circular growths and numerous satellite growth locations, while A549 cells continued to thicken the border with limited satellite growth observed.

**Fig. 5:**
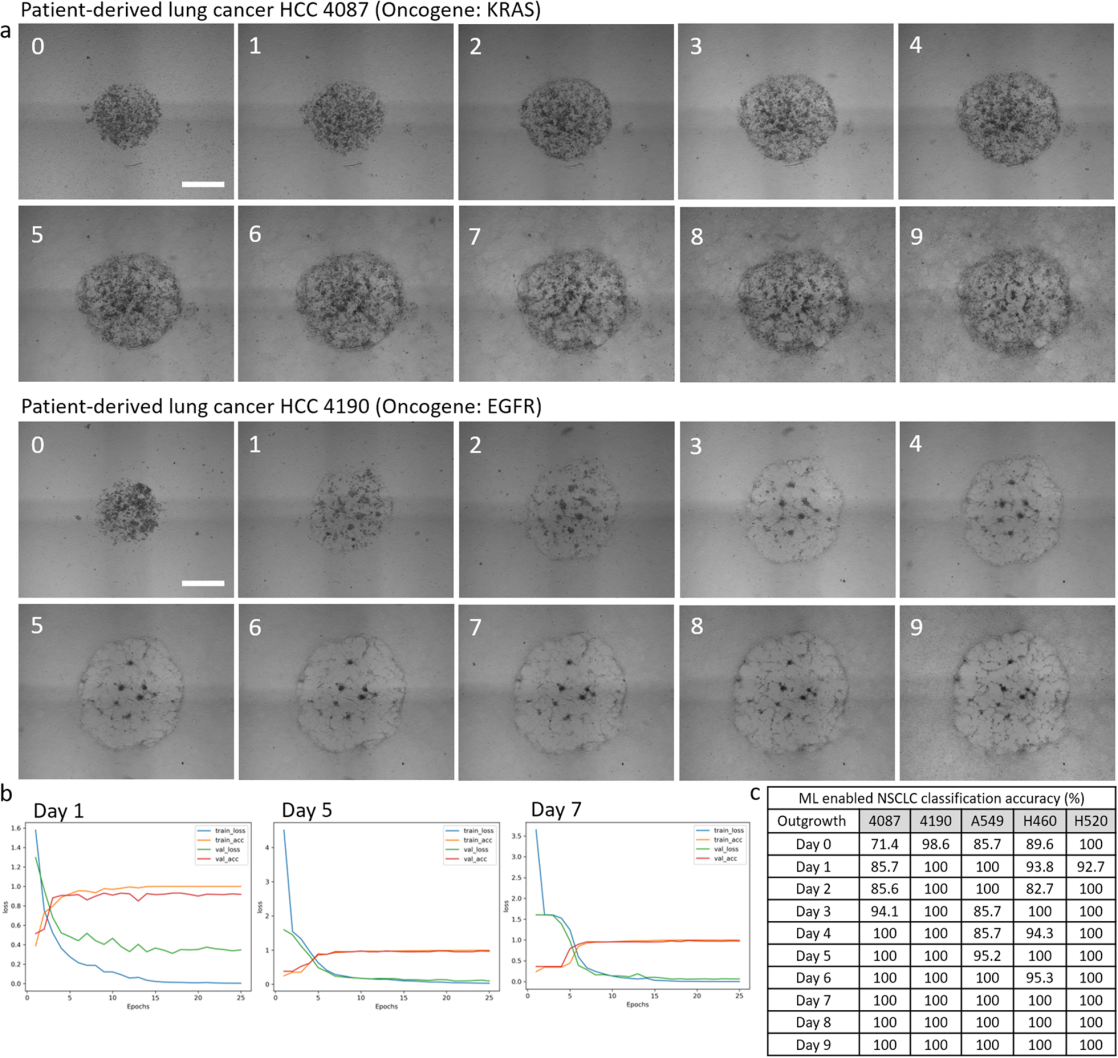
Patient-derived lung cancer cell classification. **a** Representative sequences of cancer outgrowth over surrounding fibroblasts for HCC 4087 (KRAS oncogene) and HCC 4190 (EGFR oncogene) over 9 days. Scale bar = 1 mm. **b** Classification results for all five NSCLC subtypes including two patient-derived lung cancer cells trained for 25 epochs using the lightweight TinyVGG model. All training parameters were kept consistent across all 10 sets of training. The graphs display the training loss (blue), training accuracy (orange), validation loss (green), and validation accuracy (red). The x-axis represents the number of epochs, and the y-axis indicates classification accuracy, where an accuracy of 1 corresponds to 100% correct classification. Only results for Days 1, 5, and 7 are shown. **c** Lightweight CNN-enabled NSCLC classification accuracy for Days 0–9. Validation accuracy of 1 represents 100% classification accuracy

We hypothesized that the CNN model would show gradually increasing classification accuracy by day of cancer outgrowth, likely starting near 33% (equivalent to a random guess within 3 classes: H460, A549, or H520), as human observers noticed more differences with time. Surprisingly, the lightweight CNN model achieved 86% classification accuracy for A549, 90% for H460, and 100% for H520 at Day 0 (Fig. 4b and Fig. 5c). The classification accuracy further changed to 100% for A549, 94% for H460, and 93% for H520 at Day 1. Although there were slight fluctuations in classification accuracy (ranging from 83 to 100%), the CNN model achieved 100% classification accuracy for all subtypes at 7 days of outgrowth (Fig. 5c). Interestingly, the model required more outgrowth days to achieve 100% classification accuracy for the robustly outgrowing subtypes (A549 and H460) but achieved 100% accuracy at Day 2 for the slower-growing subtype (H520).

These results suggest that the CNN model can accurately classify NSCLC subtypes even at early stages of outgrowth, with increasing accuracy as the outgrowth progresses.

### Lightweight CNN-enabled patient-derived lung cancer cell classification

We employed the lightweight CNN model to classify patient-derived lung cancer cells, both categorized as adenocarcinomas but with distinct genetic profiles: one harboring the KRAS oncogene (HCC 4087) and the other the EGFR oncogene (HCC 4190). Although these cells are classified as adenocarcinoma, their outgrowth patterns over surrounding fibroblasts varied depending on their oncogene expressions (Fig. 5a). On the seeding day (Day 0), it was challenging to distinguish them through visual inspection. However, as the cells progressed to Day 9 of outgrowth, the differences became more noticeable. HCC 4087 gradually outgrew over fibroblasts while maintaining its cellular boundary and darkening center mass, whereas HCC 4190 quickly lost its center mass but robustly infiltrated the surrounding fibroblasts. Interestingly, the classification accuracy reached 100% on Day 1 for HCC 4190, while it took until Day 4 to reach 100% classification accuracy for HCC 4087 (Figs. 5b, c and Supplementary Fig. 3). For HCC 4087, the accuracy gradually increased from 71 to 100%. The CNN model achieved 100% classification accuracy at 4 days of outgrowth for both patient-derived lung cancer cells.

These results strongly suggest that the CNN model can accurately classify patient-derived lung cancer cells even at early stages of outgrowth, demonstrating high translational potential for clinical lung cancer diagnosis.

## Discussion

In this study, we have demonstrated that a lightweight CNN model can accurately classify NSCLC subtypes, including patient-derived lung cancers, even at early stages of outgrowth, highlighting its high translational potential for clinical lung cancer classification. CNNs enhance the ability of researchers and clinicians to detect subtle patterns and features in images that are often challenging to discern through visual inspection [3, 5, 10–12]. While many sophisticated and deep neural networks exist [6–9], optimizing the input dataset for machine learning can help mitigate the need for extensive computing power. This approach broadens access to machine learning technology, allowing more researchers and clinicians to leverage these tools without investing heavily in high-performance GPUs for neural network training.

Our study employed 512 × 512 greyscale images to classify lung cancer subtypes based on their outgrowth patterns and the cellular interactions between cancerous cells and fibroblasts, which result in unique pixel darkening. The lightweight CNN model was trained on a locally operated laptop without network or cloud connectivity. Training for five NSCLC subtypes (using 2374 augmented images over 25 epochs at each outgrowth stage) took approximately 40 min, while classification accuracy was determined in about 20 s using 520 augmented images for validation. This lightweight feature enables clinicians to perform lung cancer classification on patient information-protected laptops.

However, there are challenges that need to be addressed in machine learning, such as the quality of the input data and the size of the training dataset. Many studies emphasize the importance of preparing the input dataset for optimal machine learning performance. This preparation includes labeling the dataset, cropping and resizing images, cleaning the images, and augmenting them to ensure the model can learn weights applicable to a broad range of cases [20–23].

Our study could benefit from additional testing on lighting normalization and artifact removal (e.g., bubbles, debris, or scratches in images) to ensure the CNN differentiates based on the cancer outgrowth over surrounding fibroblasts rather than unrelated features. Additionally, establishing a train model with a significantly larger number of lung cancer subtypes, including multiple variants of each subtype with various histological and genetic profiles, would enhance the model’s generalizability and robustness in classifying all lung cancer subtypes.

In summary, while our lightweight CNN model demonstrates promising results for NSCLC classification, further refinement and expansion of the training dataset are essential for improving its robustness and ensuring its broader applicability in clinical settings.

## Conclusions

In conclusion, our results demonstrate that a lightweight CNN architecture can effectively classify lung cancer subtypes based upon their outgrowth patterns in the presence of surrounding fibroblasts. Machine learning significantly expands the possibilities of diagnostic testing, enabling capabilities previously unattainable. While similar cells are challenging to differentiate at low magnification, machine learning can reliably classify lung cancer subtypes by analyzing their outgrowth patterns over fibroblasts under these conditions. This approach, which requires only low magnification with brightfield illumination, simplifies and enhances data collection efficiency. Furthermore, our findings highlight the valuable information that can be gleaned about cancer cells outgrowth on a macro-level, underscoring that there is much to learn beyond the detailed properties of cells such as morphology and gene expression.

### Supplementary Information

Supplementary file 1

Supplementary file 2

Supplementary file 3

Supplementary file 4

## Data Availability

The datasets used and/or analyzed during the current study are available from the corresponding author on reasonable request.

## Abbreviations

CNN: Convolutional neural network
SCLC: Small cell lung cancer
NSCLC: Non-small cell lung cancer
ADC: Adenocarcinoma
SCC: Squamous cell carcinoma
LCC: Large cell carcinoma
ReLU: Rectified linear unit
