# Supplementary figures and images for: Machine learning enabled classification of lung cancer cell lines co-cultured with fibroblasts with lightweight convolutional neural network for initial diagnosis
Source: J Biomed Sci. 2024 Aug 23;31:84. doi: 10.1186/s12929-024-01071-0 (PMC11344461; doi:10.1186/s12929-024-01071-0)

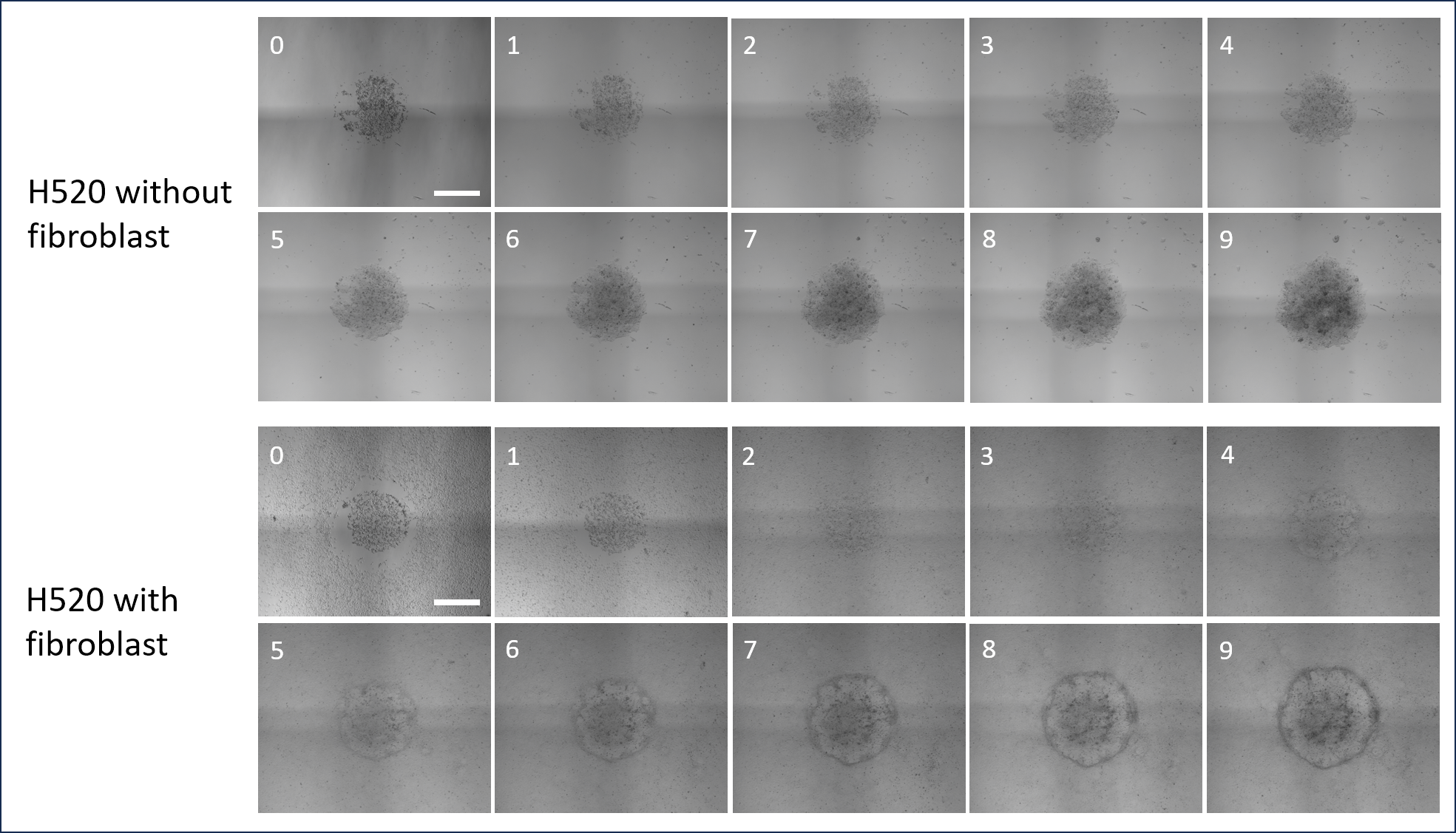

Supplement: Supplementary file 1 [file 12929_2024_1071_MOESM1_ESM.png]

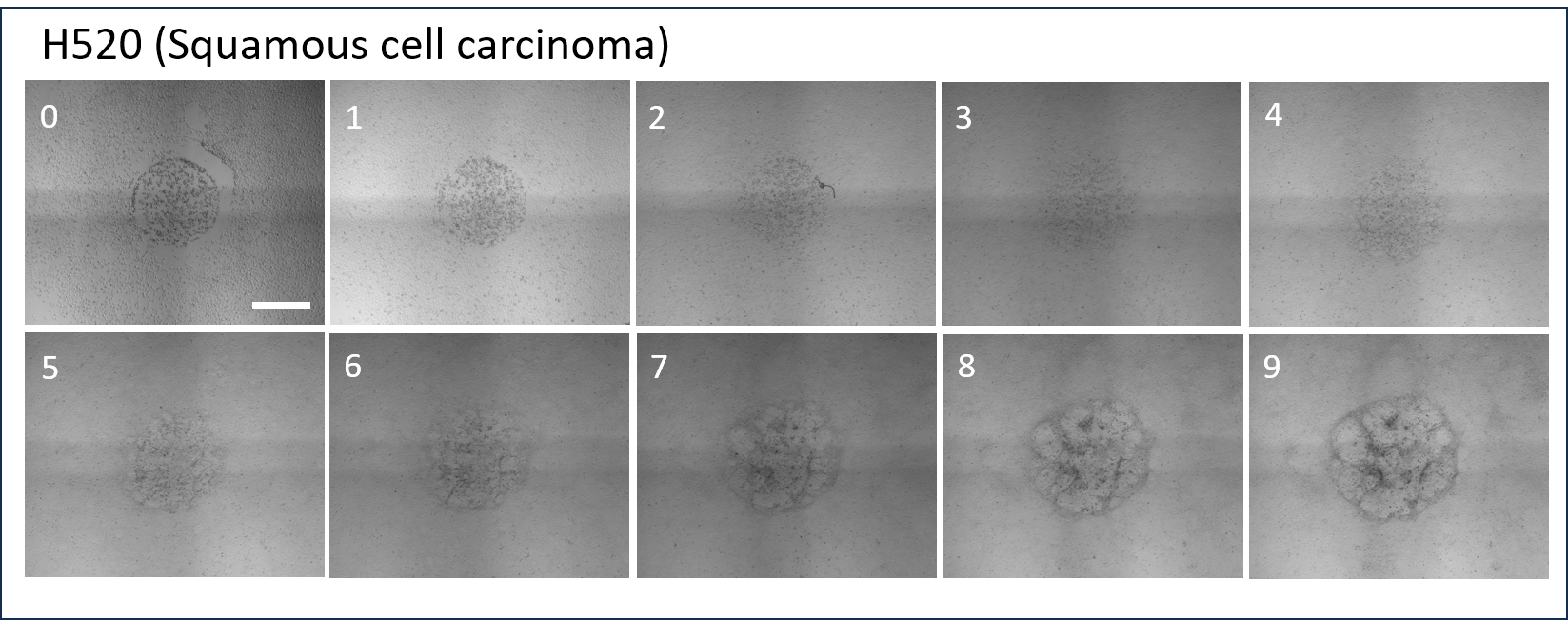

Supplement: Supplementary file 2 [file 12929_2024_1071_MOESM2_ESM.png]

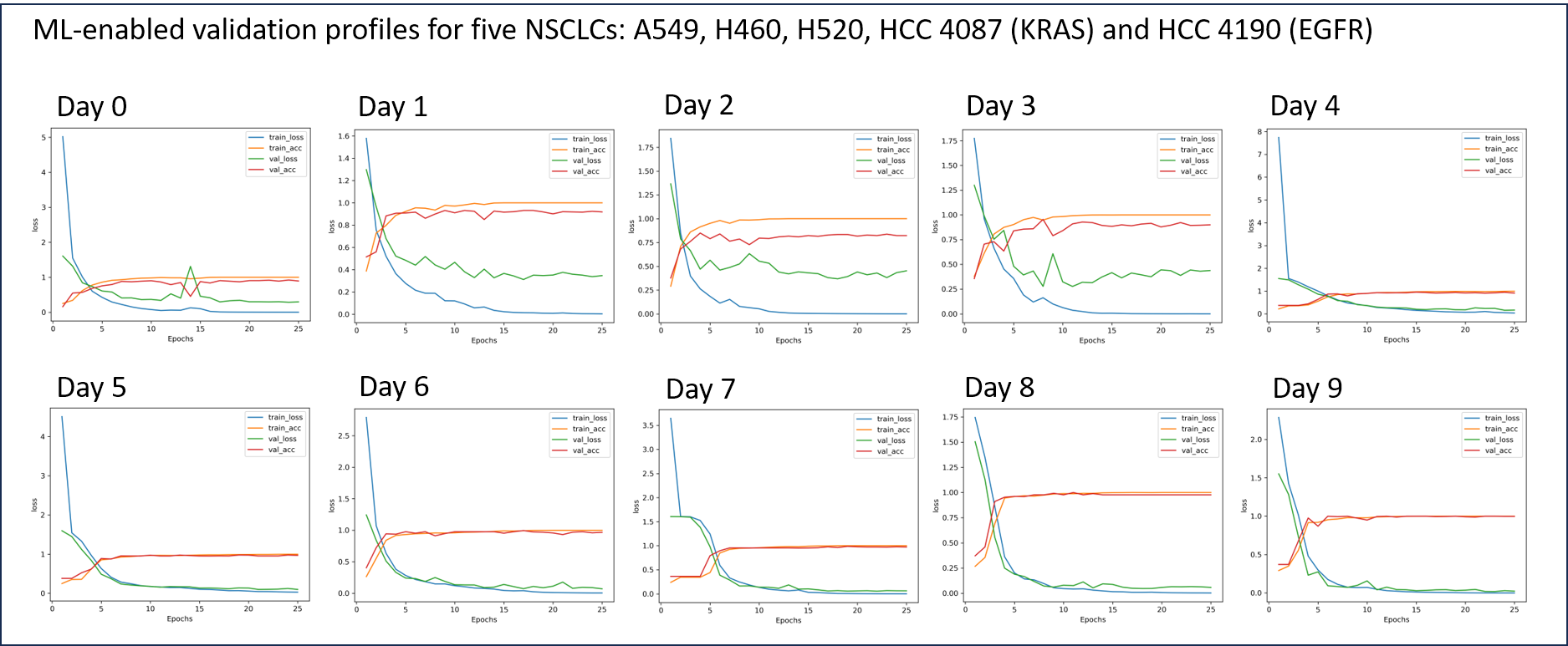

Supplement: Supplementary file 3 [file 12929_2024_1071_MOESM3_ESM.png]
