# Supplementary material for: Machine learning enabled classification of lung cancer cell lines co-cultured with fibroblasts with lightweight convolutional neural network for initial diagnosis
Source: J Biomed Sci. 2024 Aug 23;31:84. doi: 10.1186/s12929-024-01071-0 (PMC11344461; doi:10.1186/s12929-024-01071-0)
Supplement: Supplementary file 4 [file 12929_2024_1071_MOESM4_ESM.docx]

***Machine Learning enabled classification of lung cancer cell lines co-cultured with fibroblasts with lightweight convolutional neural network for initial diagnosis***

***Supplementary Figures***

Adam Germain^1^, Alex Sabol^2^, Anjani Chavali^1^, Giles Fitzwilliams^1^, Alexa Cooper^3^, Sandra Khuon^4^, Bailey Green^1^, Calvin Kong^1^, John Minna, MD^5^ and Young-Tae Kim, PhD^1,6*^

*^1^Department of Bioengineering, University of Texas at Arlington, TX*

*^2^Department of Computer Science, University of Texas at Arlington, TX*

*^3^Department of Biology, University of Texas at Arlington, TX*

*^4^Department of Nursing, University of Texas at Arlington, TX*

*^5^Hamon Center for Therapeutic Oncology Research, University of Texas Southwestern Medical Center, Dallas, TX*

*^6^Department of Urology, University of Texas Southwestern Medical Center, Dallas, TX*

Correspondence and Requests for materials should be addressed to:

*Corresponding author:

Young-Tae Kim, Ph.D.

Department of Bioengineering

500 UTA Blvd ERB244

University of Texas at Arlington

Arlington, TX 76010

E-mail : ykim@uta.edu

Fax : 817-272-2251

Phone: 817-272-5023

**Supplementary Figure 1.**  **Comparison of H520 cancer outgrowth with and without surrounding fibroblasts.** Representative sequences of H520 (squamous cell carcinoma) cancer outgrowth without surrounding fibroblasts (top) and with surrounding fibroblasts (bottom) over 9 days. 0: 3 hours after seeing both lung cancer cells at the center (and surrounding fibroblasts, only bottom). 1-9: 1-9 days after seeding. Scale bar = 1mm. H520 without surrounding fibroblasts forms a dense sphere, while H520 with surrounding fibroblasts slowly outgrows over the fibroblasts, forming a clear cellular boundary.

**Supplementary Figure 2. H520 (squamous cell carcinoma) outgrowth over surrounding fibroblasts.** Representative sequences of H520 cancer outgrowth over surrounding fibroblasts over 9 days. 0: 3 hours after seeing both lung cancer cells at the center and surrounding fibroblasts. 1-9: 1-9 days after seeding. Scale bar = 1mm.

**Supplementary Figure 3. ML-enabled validation profiles for five NSCLCs: A549, H460, H520, HCC 4087 (KRAS), and HCC 4190 (EGRF).** Classification results for all five NSCLC subtypes including two patient-derived lung cancer cells trained for 25 epochs using the lightweight TinyVGG model. All training parameters were kept consistent across all 10 sets of training (Day 0 to 9). The graphs display the training loss (blue), training accuracy (orange), validation loss (green), and validation accuracy (red). The x-axis represents the number of epochs, and the y-axis indicates classification accuracy, where an accuracy of 1 corresponds to 100% correct classification.
